# Age and Sex-Associated Changes of Complement Activity and Complement Levels in a Healthy Caucasian Population

**DOI:** 10.3389/fimmu.2018.02664

**Published:** 2018-11-20

**Authors:** Mariana Gaya da Costa, Felix Poppelaars, Cees van Kooten, Tom E. Mollnes, Francesco Tedesco, Reinhard Würzner, Leendert A. Trouw, Lennart Truedsson, Mohamed R. Daha, Anja Roos, Marc A. Seelen

**Affiliations:** ^1^Division of Nephrology, Department of Internal Medicine, University of Groningen, University Medical Center Groningen, Groningen, Netherlands; ^2^Department of Obstetrics and Gynecology, Martini Hospital, Groningen, Netherlands; ^3^Department of Nephrology, University of Leiden, Leiden University Medical Center, Leiden, Netherlands; ^4^Department of Immunology, Oslo University Hospital and University of Oslo, Oslo, Norway; ^5^Research Laboratory, Bodø Hospital, and K.G. Jebsen TREC, University of Tromsø, Tromsø, Norway; ^6^Centre of Molecular Inflammation Research, Norwegian University of Science and Technology, Trondheim, Norway; ^7^Immunorheumatology Research Laboratory, Istituto Auxologico Italiano, IRCCS, Milan, Italy; ^8^Department of Hygiene, Microbiology and Public Health, Medical University of Innsbruck, Innsbruck, Austria; ^9^Department of Rheumatology, Leiden University Medical Center, Leiden, Netherlands; ^10^Department of Immunohematology and Blood Transfusion, Leiden University Medical Center, Leiden, Netherlands; ^11^Department of Laboratory Medicine, Section of Microbiology, Immunology and Glycobiology, Lund University, Lund, Sweden; ^12^Department of Medical Microbiology and Immunology, St. Antonius Hospital, Nieuwegein, Netherlands

**Keywords:** complement, health, sex and age, innate imunity, gender

## Abstract

**Introduction:** The complement system is essential for an adequate immune response. Much attention has been given to the role of complement in disease. However, to better understand complement in pathology, it is crucial to first analyze this system under different physiological conditions. The aim of the present study was therefore to investigate the inter-individual variation in complement activity and the influences of age and sex.

**Methods:** Complement levels and functional activity were determined in 120 healthy volunteers, 60 women, 60 men, age range 20–69 year. Serum functional activity of the classical pathway (CP), lectin pathway activated by mannan (MBL-LP) and alternative pathway (AP) was measured in sera, using deposition of C5b-9 as readout. In addition, levels of C1q, MBL, MASP-1, MASP-2, ficolin-2, ficolin-3, C2, C4, C3, C5, C6, C7, C8, C9, factor B, factor D, properdin, C1-inhibitor and C4b-binding protein, were determined. Age- and sex-related differences were evaluated.

**Results:** Significantly lower AP activity was found in females compared to males. Further analysis of the AP revealed lower C3 and properdin levels in females, while factor D concentrations were higher. MBL-LP activity was not influenced by sex, but MBL and ficolin-3 levels were significantly lower in females compared to males. There were no significant differences in CP activity or CP components between females and males, nevertheless females had significantly lower levels of the terminal components. The CP and AP activity was significantly higher in the elderly, in contrast to MBL-LP activity. Moreover, C1-inhibitor, C5, C8, and C9 increased with age in contrast to a decrease of factor D and C3 levels. In-depth analysis of the functional activity assays revealed that MBL-LP activity was predominantly dependent on MBL and MASP-2 concentration, whereas CP activity relied on C2, C1-inhibitor and C5 levels. AP activity was strongly and directly associated with levels of C3, factor B and C5.

**Conclusion:** This study demonstrated significant sex and age-related differences in complement levels and functionality in the healthy population. Therefore, age and sex analysis should be taken into consideration when discussing complement-related pathologies and subsequent complement-targeted therapies.

## Introduction

The complement system, a major component of innate immunity, plays a crucial role in the immune response. In health, maintaining a balance between activation and inhibition of the complement system is key to preserve tissue homeostasis and to enable immune surveillance (1, 2). However, an overactive system can cause autoimmune and inflammatory diseases, whereas an inactive complement system results in an increased risk for infection. Several elements can disrupt this delicate balance and with age these effects are exacerbated. Complement deficiencies or dysfunctions have been shown to be associated with diseases such as atypical hemolytic uremic syndrome (aHUS), age-related macular degeneration (AMD), paroxysmal nocturnal hemoglobinuria (PNH), systemic lupus erythematosus (SLE), C3 glomerulopathy (C3G), and other kidney diseases (3, 4). Furthermore, complement activation contributes to several diseases and pathological conditions such as renal replacement therapy (5–7), cancer (8), hypersensitivity reactions (9, 10), and neurological conditions (11).

The complement system is activated via an enzymatic cascade reaction and has three different activation pathways, namely the classical pathway (CP), the lectin pathway (LP), and the alternative pathway (AP). Complement activity for each of these pathways depends on the expression and function of a large number of complement proteins. The CP is mainly initiated by immune complexes binding to C1q leading to activation of C1r and C1s, but can also be activated in an antibody independent manner like C1q binding to C-reactive protein. The LP is initiated by the binding of mannose-binding lectin (MBL), ficolins, or collectins to sugars or acetylated compounds resulting in the activation of the MBL-associated serine protease (MASP)-1 and MASP-2. Recently it has also been shown that the LP can be activated by naturally occurring antibodies, underscoring the overlap and cross-talk between the pathways (12). LP and CP activation both lead to the cleavage of C4 and C2, and subsequently to the generation of C4b2a, the C3-convertase. Furthermore, C1-inhibitor (C1-INH) regulates the activity of the recognition complexes, while C4b-binding protein (C4bp) functions as a cofactor for factor I-mediated cleavage of C4b. Initiation of the AP occurs via spontaneous hydrolysis of C3 into C3(H_2_O) or by the binding of C3b to altered surfaces. Factor D cleaves factor B when the latter is complexed with C3b, creating the C3-convertase of the AP, C3bBb. In addition, this C3-convertase is stabilized by properdin, the only positive regulator of the complement system. Regardless of the initial pathway, complement activation can lead to the initiation of the terminal pathway (TP) and thereby the generation of C5-convertase, which cleaves C5 in C5a, a powerful anaphylatoxin, and C5b. Next, C5b binds C6 which then attaches to a surface and interacts with C7, C8, and C9 to form the membrane attack complex (MAC/C5b-9) (13, 14). If there is no surface present, C5b6 will bind to C7, C8 and C9 together with the control proteins vitronectin and clusterin in the fluid-phase and thereby the soluble form of the terminal complement complex, sC5b-9, is formed. To prevent unintended complement activation the system is kept under control by a variety of regulators. The major regulators of the AP are the plasma proteins factor H and factor I. Factor H inhibits complement activation by accelerating the decay of the C3bBb convertase of the AP and by providing cofactor activity for factor I-mediated cleavage of C3b.

Both age and sex are known to influence and significantly impact the immune system (15). Females and males have distinct innate and adaptive immune responses (16). Moreover, they also differ in their immunological responses to self and foreign-antigens (17). Sex-based immunological differences can be found in various species (18). In general, the immune response seems to be stronger in females than in males (15). These immunological sex differences are thought to arise from discrepancies in hormones, genetic factors and environmental mediators. Notably, these sex-related differences in the immune system could give insight into the epidemiology and etiology of autoimmune and infectious diseases (15). Likewise, aging is associated with a progressive decline in immunity. The impact of aging on adaptive immunity is well accepted (19). However, less certainty exists on the effect of aging on innate immunity (20). Impaired function of neutrophils and macrophages as well as reduced interaction between dendritic cells and T cells, suggest also a decline in innate immune function (21). As a result of impaired immune function, the ability of elderly to respond to microorganisms is diminished and the number of infectious disease is increased (22). Also the increased incidence of autoimmune diseases might be related to altered immunity in elderly (23).

In the current era of personalized medicine combined with the recent success of complement inhibitors in clinical trials, it is essential to identify the influence of sex and age on the complement system. Given that complement therapeutics are an effective treatment for complement-mediated diseases, individuals will need different doses in order to efficiently block the complement system depending on the concentrations and functional activity of the complement components. Factors influencing the levels could be e.g., sex and age. Thus, the aim of the present study was to explore the effect of age and sex on the complement system in a healthy Caucasian population by performing functional and quantitative complement analyses. The current study also enables us to better understand the complement system in different physiological conditions.

## Materials and methods

### Serum samples

Serum samples were obtained from a population of 120 healthy Caucasian individuals from Norway, registered as blood donors. From each sex, 12 samples were obtained per age decade between 20 and 70. Sixty females with a mean age of 44.7 years (range: 20–69 years) and 60 males with a mean age of 45.1 years (range: 20–65 years) were included. Serum samples obtained were directly aliquoted and stored at −80°C.

### Assessment of pathway activity in normal human serum samples

The complement kit (Complement System Screen Wieslab®, Eurodiagnostica, Malmö, Sweden) for assessment of CP, LP, and AP activity was used according to the manufacturer's instructions (24, 25). In brief, strips of wells for CP were coated with IgM, strips for MBL-LP were coated with mannan and strips for AP were coated with LPS. Sera were diluted 1/101 for the CP and MBL-LP assays and 1/18 for the AP assay in specific buffers to ensure that activation of only the actual pathway occurred, and were incubated for 1 h at 37°C. After washing, alkaline phosphatase-conjugated anti-human C5b-9 was added before incubation at room temperature for 30 min. Additional washing was performed, substrate was added and the wells were incubated for 30 min. Finally, absorbance values were read at 450 nm. In each assay standard positive and negative control sera, provided in the kit as lyophilized material and reconstituted with distilled water, were assessed. The positive standard serum was a pool of 5 sera from healthy individuals and the negative control consisted of sera heat-inactivated at 56°C for 20 min. Complement activity was calculated using the following formula: activity = 100% x (mean A450 (sample)–mean A450 (negative control) / (mean A450 (standard serum) - mean A450 (negative control). Samples as well as standard serum and negative control serum were tested in duplicate.

### Serum complement concentrations

Assessment of MBL concentrations was performed using a commercial MBL ELISA obtained from BIOPORTO Diagnostics A/S (Hellerup, Denmark) and performed according to the manufacturer's instructions. C1q, C4, C3, and C1-inhibitor concentrations were assessed using nephelometry on a BN-Prospec, with validated diagnostic protocols provided by the manufacturer (Siemens Healthcare Diagnostics, Marburg, Germany), calibrated using a complement standard serum provided by the manufacturer. C2 and factor B were measured using rocket-immunoelectrophoresis as described by Sjöholm et al. (26–28). Properdin and Factor D were assessed by electroimmunoassay as previously described (29, 30). Concentrations of C2, factor B, properdin and factor D were expressed in arbitrary units (% of pooled human serum).

MASP-1, MASP-2, Ficolin-2, and Ficolin-3 levels were determined using in-house ELISAs. For all in house ELISA's (Leiden University Medical Center, Leiden, the Netherlands) a standard protocol was used. In brief, Nunc Maxisorb plates (Nunc, Roskilde, Denmark) were coated using coating buffer (100 mM Na2CO3/NaHCO3, pH 9.6), for 16 h at room temperature. After each step, plates were washed three times with PBS containing 0.05% Tween 20. Residual binding sites were blocked by incubation with PBS containing 1% BSA. Unless otherwise indicated, all subsequent steps were incubated in PBS containing 0.05% Tween 20 and 1% BSA, for 1 h at 37°C. Detection antibodies were conjugated to digoxigenin (Dig) using Dig-3-*O*-methylcarbonyl-ε-aminocaproic acid-N-hydroxysuccinimide ester (from Boehringer Mannheim, Mannheim, Germany), followed by detection using HRP-conjugated rabbit anti-Dig Abs (Fab, from Boehringer Mannheim). Enzyme activity of HRP was detected using 2,2′-azino-bis(3-ethylbenzthiazoline-6-sulfonic acid) (Sigma) and absorption was measured at 415 nm.

In more detail, for the detection of MASP-1 and MASP-2, polyclonal antibodies were raised in rabbits against the recombinant protease domain of MASP-1 and MASP-2, respectively, kindly provided by Dr. Peter Gál (31). However, based on the specificity of the anti-MASP-1 antibody it is likely that the results from this ELISA represent the sum of MASP-1 and MASP-3 levels. ELISA plates were coated with rabbit IgG anti-MASP-1 and rabbit IgG anti-MASP-2, respectively. Patient samples were incubated in GVB/NaCl/EDTA (Veronal-buffered saline containing 0.05 % Tween-20, 0.1 % gelatine, 0.5M NaCl, 10 mM EDTA; pH 7.5), followed by detection using the same antibodies, which were conjugated as described above. Results were expressed in arbitrary units per ml using a standard line of serially diluted pooled normal human serum for calibration. For detection of ficolin-2, a similar protocol was followed using mouse monoclonal antibody GN5. Results were expressed in pg/ml as described previously (32). Both GN5 and 4H5 were kindly provided by dr. T. Fujita, Department of Immunology, Fukushima Medical University, Fukushima, Japan). For detection of ficolin-3, the standard ELISA protocol was followed and the mouse monoclonal antibody 4H5 was used for both coating and detection. Results were expressed in arbitrary units/ml using pooled human serum for calibration.

C4bp, C5, C8, and C9 were measured by ELISA as previously described (33–35). C6 and C7 were measured using an in-house ELISA assay as previously described (36). In brief, wells were coated with an in house mouse monoclonal antibody against C6 (WU 6-4, Hycult, Uden, NL) and with an in house polyclonal antibody against C7. For the detection, respective biotinylated polyclonal antibodies were used. For the set-up of the ELISA, purified C6 or C7 (Cytotech, San Diego, CA, USA) were used to calibrate human serum as a standard.

The standards used in the different assays were not calibrated to the official European complement standard.

### Statistics

Statistical analysis was performed using IBM SPSS 22.0 (IBM Corporation, Chicago, IL, USA). Concentrations of complement proteins and pathway activity were presented as median with interquartile range [IQR]. Differences between sexes were assessed with the Mann Whitney U test. Correlation between age and complement proteins or pathway activity was evaluated using the Spearman Rank correlation coefficient (*r*). Univariate and subsequent multivariate linear regression analysis were performed to identify independent determinants of complement pathway activity. Multivariate analysis models were constructed using backward selection (*P*_out_ > 0.05) including complement proteins that were associated significantly (*P* < 0.05) with complement pathway activity in univariate analysis. *P* < 0.05 were considered statistically significant.

## Results

### Differences between males and females in complement pathway activity and components

**Figure 1:**
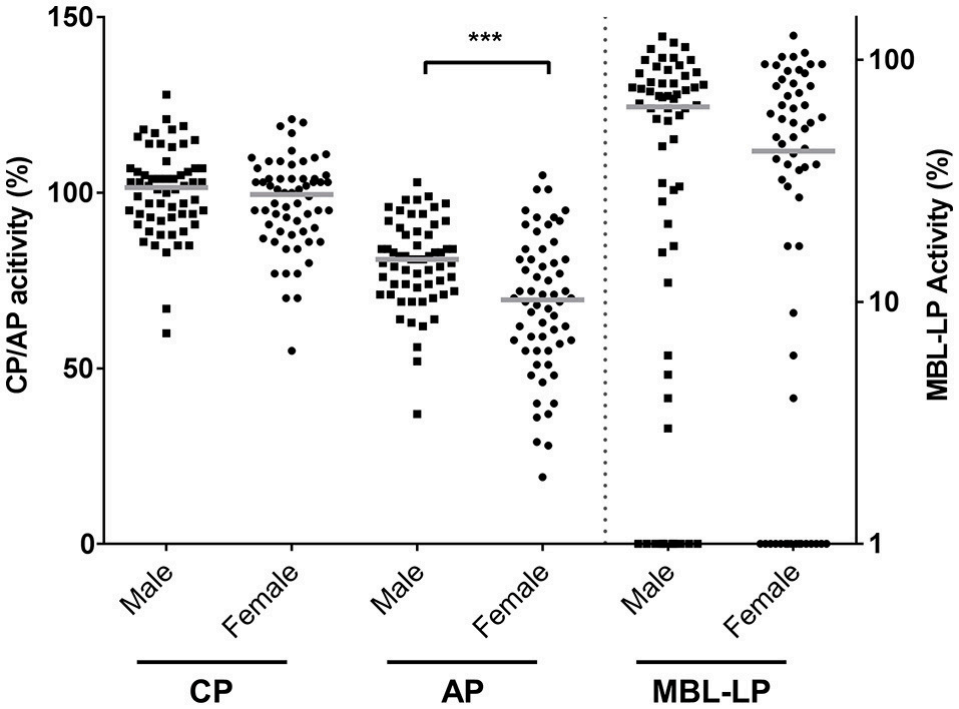
Complement pathway activity according to sex. The activity of the classical pathway (CP), alternative pathway (AP), and MBL-induced lectin pathway (MBL-LP) was measured in 120 Caucasian healthy subjects, of which 60 males and 60 females. The solid lines indicate the median values in each group. The differences between males and females was assessed by the Mann Whitney test (***P < 0.001). CP/AP activity is referred to the left Y-axis in linear scale whereas MBL-LP activity is referred to the right Y-axis in a logarithm scale.

CP, MBL-LP, and AP activity were determined by deposition of C5b-9 in serum samples from 60 males and 60 females. CP and MBL-LP activity were similar in both sexes (Figure 1). However, AP activity was significantly lower in females compared to males. The median AP activity for females was 69.5% compared with 81.0% for males (*P* < 0.001, Figure 1).

**Figure 2:**
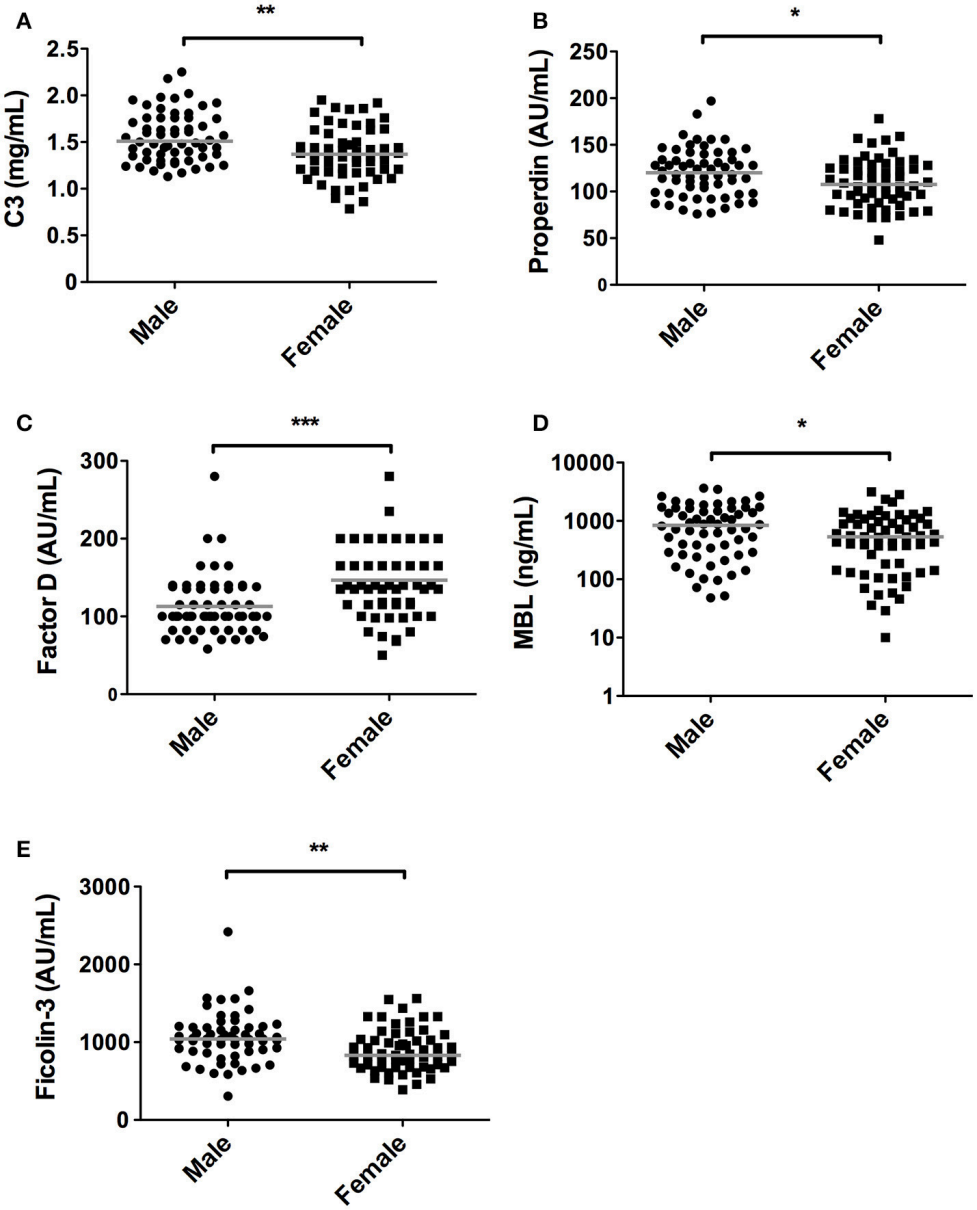
Differences in complement components between sexes. In 120 subjects, consisting of 60 males and 60 females complement levels of **(A)** C3, **(B)** Properdin, **(C)** Factor D, **(D)** MBL, **(E)** Ficolin-3 were measured. The solid lines indicate the median values in each group. The differences between males and females was assessed by the Mann Whitney test (*P < 0.05, **P < 0.01, ***P < 0.001).

In accordance, the levels of C3 and properdin were also significantly lower in women in comparison with men. Median C3 concentrations in females were 1.37 mg/mL [1.19–1.59] compared to 1.51 mg/mL [1.34–1.76] in males (*P* = 0.001, Figure 2A), whereas median levels of properdin in females were 107% [91–128] vs. 120% [98–138] in males (*P* = 0.03, Figure 2B). Conversely, serum concentration of factor D showed an opposite pattern (*P* < 0.001, Figure 2C), since levels were significantly higher in females (140%, IQR: 115%−200%) than in males (100%, IQR: 82%−137%). Males had higher levels of serum MBL in relation to female subjects. Median MBL concentrations were 533 ng/mL [142–1,076] and 843 ng/mL [289–1,646] in females and males, respectively (*P* = 0.03, Figure 2D). Likewise, female subjects also had significantly lower levels of ficolin-3 compared to male subjects, with a median of 830 AU/mL [676–1,034] and 1042 AU/mL [883–1,192] in females and males, respectively (*P* = 0.001, Figure 2E).

The rest of the components measured did not differ between females and males (Table 1). In line with CP activity, serum levels of C1q, C4, C2, C1-INH, and C4bp did not differ between the sexes (Table 1). Furthermore, despite the fact that MBL-LP activity was not significantly different between the sexes, a trend was still seen for lower activity in females compared to males. Additionally, 17% of the males were MBL deficient (0% MBL-LP activity) whereas in females this was 23%. The threshold of MBL concentration to result in zero activity was 130 ng/mL. The other components from the LP such as MASP-1, MASP-2 and Ficolin-2 did not differ between both sexes (Table 1).

**Table 1:** Levels of complement components that did not significantly differ between the sexes.

**Component**	**Male**	**Female**	***P*-value**
C1q (in mg/L)	141 [1.28–1.49]	136 [125–142]	0.12
C1-INH (in mg/mL)	0.28 [0.25–0.30]	0.26 [0.23–0.30]	0.07
Ficolin-2 (in pg/mL)	1680 [1160–2235]	1568 [1063–2403]	0.64
MASP-1 (in AU/mL)	700 [603–906]	749 [611–919]	0.53
MASP-2 (in AU/mL)	856 [650–1620]	799 [609–1121]	0.14
C2 (in AU/mL)	126 [104–140]	123 [107–134]	0.58
C4 (in mg/mL)	0.28 [0.20–0.32]	0.23 [0.19–0.29]	0.07
C4bp (in μg/mL)	214 [197–248]	206 [177–245]	0.30
Factor B (in AU/mL)	103 [93–118]	98 [88–120]	0.22
C6 (in μg/mL)	44.6 [31.8–65.6]	37.9 [25.3–54.9]	0.08

*The current table displays levels of components that were not significantly different. Values are presented as median and interquartile range [IQR]. P-values represent the difference between males and females tested by Mann Whitney test. Abbreviations: C1-INH, C1-inhibitor; MASP, MBL-associated serine protease; C4bp, C4b-binding protein. The standards used in the different assays were not calibrated to the official European complement standard*.

**Figure 3:**
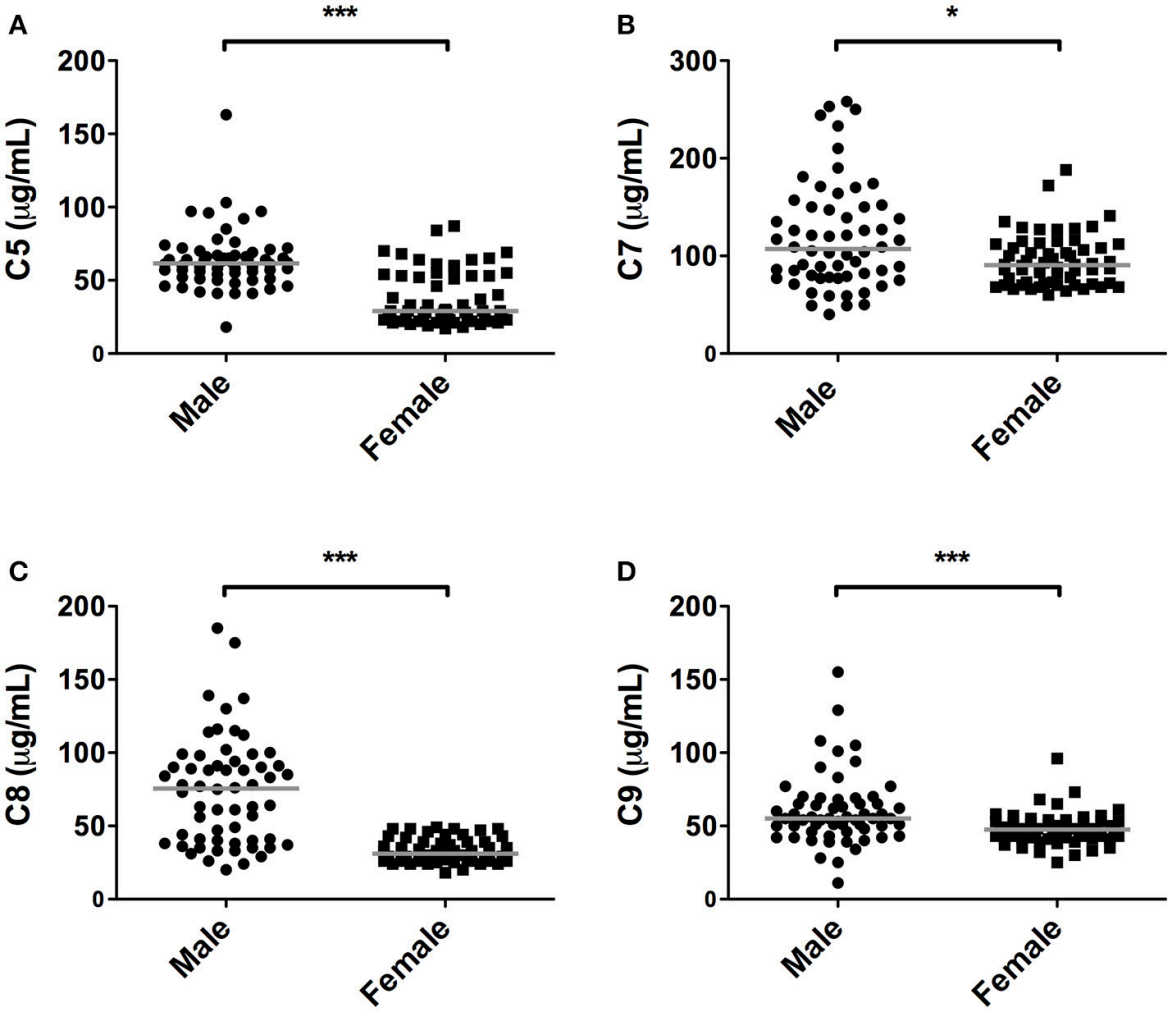
Differences in terminal pathway components between sexes. In 120 subjects, consisting of 60 males and 60 females, complement levels of **(A)** C5, **(B)** C7, **(C)** C8, and **(D)** C9 were measured. The solid lines indicate the median values in each group. The differences between males and females was assessed by the Mann Whitney test (*P < 0.05, ***P < 0.001).

Quantification of the terminal complex components revealed that all these components, except C6, were significantly lower in female subjects compared to male subjects (*P* < 0.05, Figure 3). The presented data show that in women, levels were 53, 15, 59, and 14% lower, for C5, C7, C8, and C9, respectively. For C8, concentrations above 50 μg/ml were exclusively observed in males (65% of male subjects had concentrations of C8 above 50 μg/ml).

### Age-related changes in complement pathway activity and components

**Figure 4:**
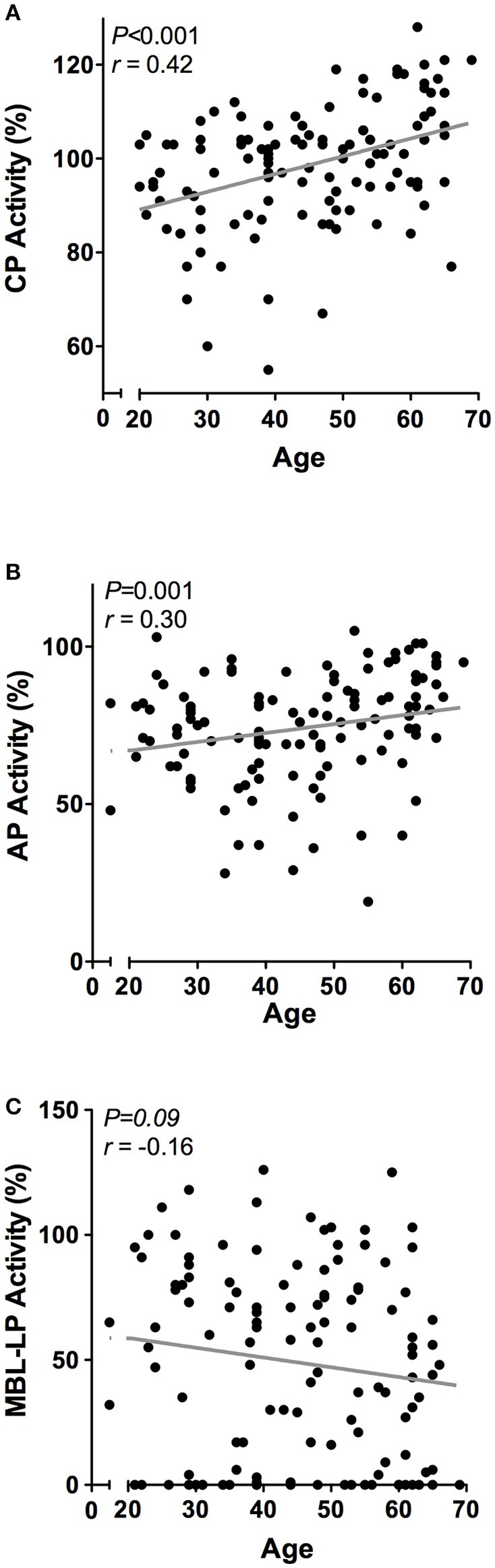
Correlations between complement pathway activity and age. In 120 healthy subjects age was correlated to the activity of the **(A)** classical pathway (CP), **(B)** alternative pathway (AP) and **(C)** MBL-induced lectin pathway (MBL-LP). These correlations were evaluated using the Spearman Rank correlation coefficient. *P* < 0.05 were considered to be statistically significant.

Next, we determined the effect of age on the complement system by correlating complement components and activity with age in 120 healthy individuals with a mean age of 45 years ± 13.5, ranging from 20 to 69 years old. Linear regression analysis demonstrated a significant age-related effect for CP and AP activity, but not for the MBL-LP activity (data not shown). In accordance, age significantly correlated with CP activity (*r* = 0.42, *P* < 0.001, Figure 4A) and AP activity (*r* = 0.30, *P* < 0.001, Figure 4B), but not with MBL-LP activity (Figure 4C).

**Figure 5:**
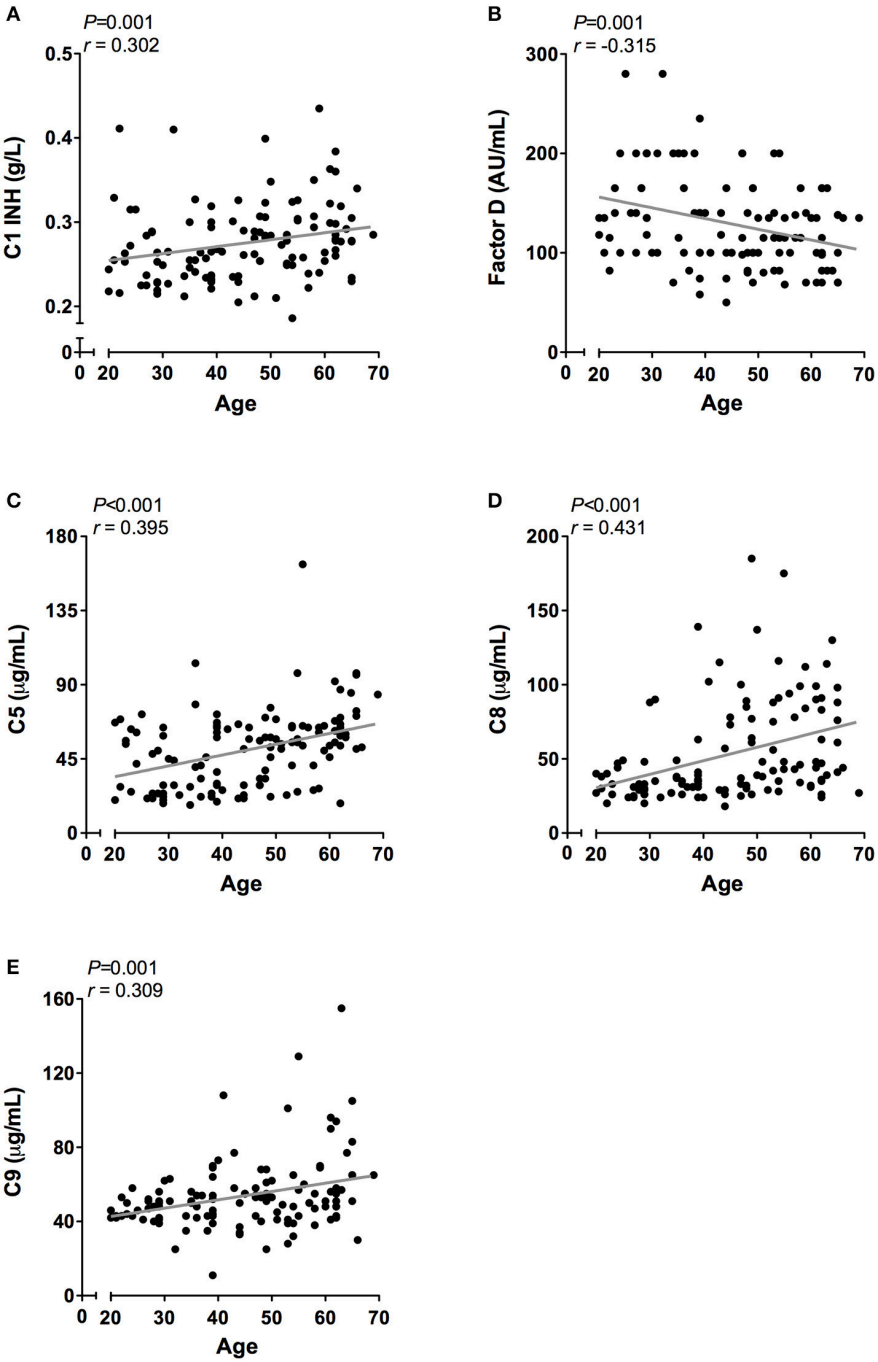
Correlation of complement proteins with age. In 120 healthy subjects age was correlated to the concentration of **(A)** C1-INH, **(B)** Factor D, **(C)** C5. **(D)** C8, and **(E)** C9. These correlations were evaluated using the Spearman Rank correlation coefficient. *P* < 0.05 were considered to be statistically significant.

However, when the twenty-five oldest individuals (mean age 62 ± 2.3 years) were compared to the twenty-five youngest individuals (mean age 26 ± 3.3 years) significant differences were observed in all pathways: higher CP activity (106 vs. 91%, *P* < 0.001), lower MBL-LP activity (35 vs. 60%, *P* = 0.01) and higher AP activity (83 vs. 74%, *P* = 0.01). Subsequently, we analyzed the effect of age on the concentration of the different complement components and factors. Regardless of statistics, a correlation coefficient below 0.3 was not perceived as clinically relevant (37). A significant effect of age was observed for C1-INH, factor D, C5, C8, and C9 levels (Figure 5). C1-INH showed an age-related rise (Figure 5A, *r* = 0.30, *P* = 0.001), whereas factor D (Figure 5B, *r* = −0.32, *P* = 0.001) levels decreased with age. Consistent with the age-related increase in CP and AP activity, levels of C5 (Figure 5C, *r* = 0.40, *P* < 0.001), C8 (Figure 5D, *r* = 0.43, *P* < 0.001) and C9 (Figure 5E, *r* = 0.31, *P* = 0.001) positively correlated with age. The comparison between the oldest and youngest twenty-five individuals showed that C5, C8, and C9 levels increased by 47% (*P* < 0.001), 60% (*P* < 0.001), and 24% (*P* = 0.001), respectively, with age. Finally, if we correct for sex, the impact of age on CP and AP activity remains significant. However, the age-related effect on AP is predominantly found in female subjects (females: *r* = 0.35, *P* = 0.05), whereas the effect of age on CP activity was predominantly found in male subjects (males: *r* = 0.61, *P* < 0.001).

### The relationship between complement components and pathway activity

**Figure 6:**
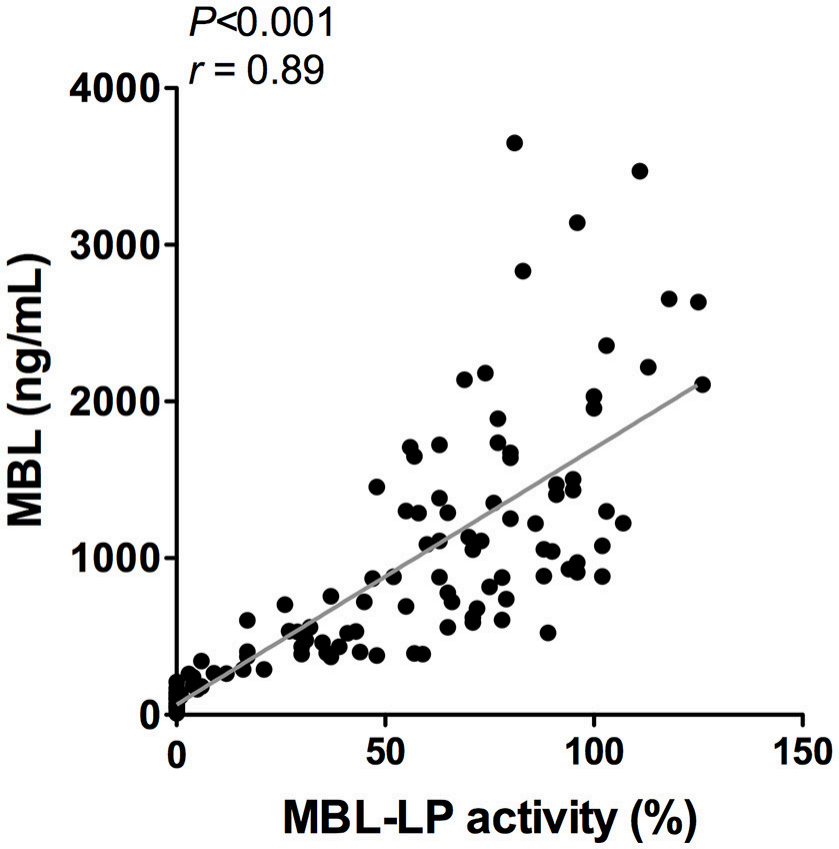
Correlation between MBL-LP activity and MBL levels. In 120 healthy subjects MBL levels were correlated to the activity of the MBL-LP (%). This correlation was evaluated using the Spearman Rank correlation coefficient. A *p* < 0.05 was considered to be statistically significant.

To better understand the determinants of complement activity of the different pathways, we related individual complement components with their respective complement pathway (Tables 2–**4**). CP activity correlated significantly with C2 levels (Table 2, *r* = 0.51, *P* < 0.001), whereas MBL-LP activity significantly correlated with MBL concentrations (Figure 6, Table 3, *r* = 0.89, *P* < 0.001). Furthermore, for AP activity significant correlations were seen with properdin (Table 4, *r* = 0.35, *P* < 0.001), factor B (Table 4, *r* = 0.51 *P* < 0.001) and C3 (Table 4, *r* = 0.42, *P* < 0.001). In addition, we correlated terminal pathway components with complement activity of the different pathways (Table 5). CP activity correlated significantly with C5 (Table 5, *r* = 0.39, *P* < 0.001) and C9 (Table 2, *r* = 0.42, *P* < 0.001), while AP activity correlated with C5 (Table 5, *r* = 0.56, *P* < 0.001), C8 (Table 5, *r* = 0.43, *P* < 0.001) and C9 (Table 5, *r* = 0.38, *P* < 0.001). MBL-LP activity did not correlate with any of the terminal pathway components (Table 5). However, besides the correlation between individual complement components with their respective complement pathway, there were also correlations between different complement components with each other. The latter could form a possible confounder for the pathway analysis. Subsequently, we performed a multivariate linear regression analysis and found that levels of C2, C1-INH, and C5 were independent determinants of CP activity (model *R*^2^ = 0.46, Table 6), whereas MBL and MASP-2 were independent determinants of MBL-LP activity (model *R*^2^ = 0.66, Table 6). Moreover, the multivariate linear regression analysis demonstrated that only C3, factor B and C5 were independent determinants of AP activity (model *R*^2^ = 0.46, Table 6).

**Table 2:** Correlations between classical pathway functional activity, complement levels, and age.

	**CP**	**C1q**	**C2**	**C4**	**C3**	**C1-INH**	**Age**
CP	–	–	–	–	–	–	0.42
							*P* < 0.001
C1q	0.20	–	–	–	–	–	−0.04
	*P* = 0.03						*P* = 0.67
C2	0.51	0.30	–	–	–	–	0.28
	*P* < 0.001	*P* = 0.001					*P* = 0.002
C4	0.11	0.29	0.32	–	–	–	0.07
	*P* = 0.27	*P* = 0.002	*P* = 0.001				*P* = 0.47
C3	0.20	0.37	0.45	0.65	–	–	0.001
	*P* = 0.03	*P* < 0.001	*P* < 0.001	*P* < 0.001			*P* = 0.99
C1-INH	0.24	0.23	0.52	0.44	0.42	–	0.30
	*P* = 0.009	*P* = 0.01	*P* < 0.001	*P* < 0.001	*P* < 0.001		*P* = 0.001
C4bp	0.24	0.28	0.44	0.29	0.39	0.38	0.13
	*P* = 0.009	*P* = 0.002	*P* < 0.001	*P* = 0.002	*P* < 0.001	*P* < 0.001	*P* = 0.17

*Spearman's correlation was performed and data are presented as correlation coefficient and corresponding P-value. Significant correlations are highlighted. C1-INH, C1-inhibitor; CP, classical pathway activity; C4bp, C4b-binding protein*.

**Table 3:** Correlations between MBL-lectin pathway functional activity, complement levels, and age.

	**MBL-LP**	**MBL**	**MASP-2**	**MASP-1**	**C2**	**C4**	**C3**	**C4bp**	**Age**
MBL-LP	–	–	–	–	–	–	–	–	−0.16
									*P* = 0.09
MBL	0.89	–	–	–	–	–	–	–	−0.16
	*P* < 0.001								*P* = 0.09
MASP-2	0.21	0.09	–	–	–	–	–	–	−0.03
	*P* = 0.025	*P* = 0.32							*P* = 0.72
MASP-1	0.09	0.06	0.29	–	–	–	–	–	−0.20
	*P* = 0.33	*P* = 0.53	*P* = 0.001						*P* = 0.03
C2	−0.08	−0.07	−0.06	0.06	–	–	–	–	0.28
	*P* = 0.39	*P* = 0.44	*P* = 0.56	*P* = 0.52					*P* = 0.002
C4	−0.06	−0.07	0.05	−0.01	0.32	–	–	–	0.07
	*P* = 0.53	*P* = 0.46	*P* = 0.63	P0.92	*P* = 0.001				*P* = 0.47
C3	−0.05	0.12	0.13	0.14	0.45	0.65	–	–	0.001
	*P* = 0.54	*P* = 0.19	*P* = 0.17	*P* = 0.12	*P* < 0.001	*P* < 0.001			*P* = 0.99
C4bp	−0.08	−0.02	0.18	−0.04	0.44	0.29	0.39	–	0.13
	*P* = 0.38	*P* = 0.83	*P* = 0.05	*P* = 0.66	*P* < 0.001	*P* = 0.002	*P* < 0.001		*P* = 0.17
C1-INH	0.08	0.15	0.03	−0.05	0.52	0.44	0.42	0.38	0.30
	*P* = 0.12	*P* = 0.12	*P* = 0.77	*P* = 0.59	*P* = < 0.001	*P* < 0.001	*P* < 0.001	*P* < 0.001	*P* = 0.001

*Spearman's correlation was performed and data are presented as correlation coefficient and corresponding P-value. Significant correlations are highlighted.C1-INH, C1-inhibitor; C4bp, C4b-binding protein; MBL, mannose-binding lectin; MBL-P, MBL-induced pathway activity; MASP, MBL-associated serine protease*.

**Table 4:** Correlations between alternative pathway functional activity, complement levels, and age.

	**AP**	**Properdin**	**Factor B**	**Factor D**	**Age**
AP	–	–	–	–	0.30
					*P* = 0.001
Properdin	0.35	–	–	–	−0.01
	*P* < 0.001				*P* = 0.89
Factor B	0.51	0.24	–	–	0.27
	*P* < 0.001	*P* = 0.01			*P* = 0.003
Factor D	0.13	−0.02	0.05	–	−0.32
	*P* = 0.17	*P* = 0.85	*P* = 0.59		*P* = 0.001
C3	0.42	0.52	0.50	0.06	0.001
	*P* < 0.001	*P* < 0.001	*P* < 0.001	*P* = 0.48	*P* = 0.99

*Spearman's correlation was performed and data are presented as correlation coefficient and corresponding P-value. Significant correlations are highlighted. AP, alternative pathway activity*.

**Table 5:** Correlations between terminal pathway components, functional pathway activity and age.

	**AP**	**CP**	**MBL-LP**	**C5**	**C6**	**C7**	**C8**	**Age**
AP	–	–	–	–	–	–	–	0.30
								*P* = 0.001
CP	0.44	–	–	–	–	–	–	0.42
	*P* < 0.001							*P* < 0.001
MBL-LP	0.02	0.04	–	–	–	–	–	−0.16
	*P* = 0.87	*P* = 0.69						*P* = 0.09
C5	0.56	0.39	0.06	–	–	–	–	0.40
	*P* < 0.001	*P* < 0.001	*P* = 0.51					*P* < 0.001
C6	0.07	−0.03	−0.05	0.18	–	–	–	0.18
	*P* = 0.44	*P* = 0.74	*P* = 0.61	*P* = 0.04				*P* = 0.04
C7	−0.09	−0.05	−0.01	0.10	0.32	–	–	0.04
	*P* = 0.34	*P* = 0.57	*P* = 0.94	*P* = 0.28	*P* < 0.001			*P* = 0.65
C8	0.43	0.27	0.00	0.59	0.20	0.22	–	0.43
	*P* < 0.001	*P* = 0.003	*P* = 0.99	*P* < 0.001	*P* = 0.02	*P* = 0.01		*P* < 0.001
C9	0.29	0.42	−0.07	0.40	0.12	0.04	0.48	0.31
	*P* = 0.001	*P* < 0.001	P = 0.47	*P* < 0.001	P = 0.18	P = 0.65	*P* < 0.001	P = 0.001

*Spearman's correlation was performed and data are presented as correlation coefficient and corresponding P-value. Significant correlations are highlighted. AP, alternative pathway activity, CP, classical pathway activity; MBL-LP, Mannose-Binding Lectin-induced pathway activity*.

**Table 6:** Univariate and multivariate determinants of functional complement pathway activity.

	**Univariate**		**Multivariate**	
	**Standardized** β	***P*****-value**	**Standardized** β	***P*****-value**
**ALTERNATIVE PATHWAY ACTIVITY**				
Factor B	0.48	< 0.001	0.28	< 0.001
Properdin	0.35	< 0.001	
C3	0.50	< 0.001	0.26	0.001
C5	0.51	< 0.001	0.37	< 0.001
C8	0.36	< 0.001	
C9	0.35	< 0.001	
**CLASSICAL PATHWAY ACTIVITY**				
C1q	0.20	0.04	
C2	0.63	< 0.001	0.62	< 0.001
C3	0.19	0.04	
C1-INH	0.20	0.03	−0.14	0.04
C4bp	0.24	0.01	
C5	0.39	< 0.001	0.25	0.001
C8	0.22	0.02	
C9	0.30	0.001	
**MBL PATHWAY ACTIVITY**				
MBL	0.79	< 0.001	0.78	< 0.001
MASP-2	0.23	0.01	0.18	0.001

*Multivariate linear regression analysis was performed to identify independent determinants of complement pathway activity. All variables described in Tables 2–5 were tested, only variables with P < 0.05 in the univariate analysis are shown. Multivariate analysis models were constructed using backward selection (P_out_ > 0.05) including complement proteins that were associated significantly (P < 0.05) with complement pathway activity in univariate analysis. C1-INH, C1-inhibitor; C4bp, C4b-binding protein; MBL, Mannose-Binding Lectin; MASP-2, MBL-associated serine protease 2*.

## Discussion

In the current study, we demonstrate in a healthy Caucasian population that sex and age significantly impact the complement system. Sex-related analysis revealed that females have lower AP activity and lower AP and LP complement components, with an exception for factor D. In addition, females showed significantly lower levels of C3 and terminal pathway components. These results demonstrate that females have significantly lower complement activity and levels of complement components compared to males. Furthermore, age-related analysis showed that aging is associated with an enhanced functional activity of the CP and the AP. Correspondingly, terminal pathway components levels increased with age. Lastly, we analyzed the determinants of functional complement pathway activity, by relating individual complement components with their respective complement pathway. In healthy individuals, CP activity was determined by C2, C1-INH, and C5, MBL-LP activity by MBL and AP activity by C3, factor B and C5. These results support the relevance of age- and sex-matched control cohorts in studies related to the complement field, including the assessment of reference values used in clinical laboratory diagnostics. Moreover, these results suggest that age and sex should be taken into account in complement-related pathology as well as in complement-targeted therapies.

For the majority of complement components the liver is the predominant source of production, and production of complement proteins can be regulated by an acute phase response (38). Other main production sites includes peripheral blood mononuclear cells which are responsible for the production of C1q, properdin and C7, adipocytes which produce factor D and the lungs where ficolin-3 is mainly produced (39, 40). In fact, most tissues and inflammatory cells are able to produce various complement proteins, e.g., upon stimulation with cytokines (40). Additionally, genetic environmental and lifestyle factors such as obesity and smoking also influence complement levels (41–43). Multiple complement deficiencies have been described and associated with pathology (44). In addition, single-nucleotide polymorphisms (SNP) can strongly affect the concentration and/or function of various complement proteins, as have been well-documented for e.g., MBL (45, 46). Yet, limited studies have investigated the influence of sex and age on the complement system (47–49).

The immune system varies between males and females and differences in innate and adaptive immunity have already been demonstrated. At least two factors are known to explain the influences of sexual dimorphism in immunity: genetics (e.g., the X chromosome) and hormonal differences (50). Remarkably, the X chromosome contains genes that encode for several proteins related to immune response such as toll-like receptors and interleukins. Moreover, properdin is encoded on the short arm of the X chromosome (51). However, in our study, females had lower properdin levels than males, demonstrating once more that genetic factors do not solely determine the concentration of the components. In addition, sex hormones are known to influence innate and adaptive immunity. The majority of the cells from innate and adaptive immunity express estrogen receptors (52). However, data on the influence of sex on the complement system remains limited. A study by Roach et al. demonstrated that in children (age range 1–19 years) there were differences in several complement components between boys and girls (49). However, the differences found in this study were age-dependent. Troldborg et al. studied complement proteins restricted to the lectin pathway and showed comparable results to ours, showing that female have lower complement levels than males (53). Previously, an animal study demonstrated the influence of sex hormones on the complement system by injecting estrogen and testosterone in healthy and castrated mice from both sexes (54). Mice treated with testosterone showed increased late acting complement activity while mice treated with estrogen showed diminished activity (48). In accordance, a recent animal study demonstrated lower complement functionality in female mice due to lower levels of terminal complement components (47). This has been a reason for our group to perform complement-related animals experiments solely in male rodents (55, 56). Altogether, these results in rodents are in line with our findings that females have lower terminal complement components and lower functional activity. In our study, only Factor D was significantly higher in woman. A possible explanation could be a higher amount of adipose tissue in woman than in men, resulting in enhanced production. Unfortunately, factor H and factor I were not determined in our cohort. Previous studies did not find significant differences between sexes in factor H levels in adults (57). In addition, very recent work did not observe an effect of sex or age in factor H levels in children (54). However, since we observed a lower AP functional activity in females, a possible explanation for this difference could be higher levels of factor H and/or factor I. Nonetheless, the clinical consequences of these differences remain to be elucidated. Yet, some clues already indicate that sex could form a possible confounder in complement-mediated diseases. For instance, in a cohort of healthy people, low MBL levels were associated with cardiovascular disease, however this association was seen only in men and not in women (58). Moreover, in a study of AMD, distinct alterations were shown between the sexes in levels of AP components (59). Thus, more studies are needed to clarify the significance of sexual dimorphism on the complement system. In the future, these shortcomings could be addressed by studying the complement system in transsexual subjects undergoing hormonal replacement therapy. Nevertheless, an important question that remains is: should we treat women and men equally when it comes to complement therapeutics?

In the early phase of life, innate immunity plays a fundamental role since adaptive immunity is still under development, whereas during adolescence this is reversed (60). Immunity undergoes severe deterioration with age (15, 60). Previous studies showed that both for innate and adaptive immunity, the function reduces with age (22, 61). Whether this decline in innate immune function is also true for the complement system was still unknown. In a Japanese healthy cohort, C3 levels varied according to different age ranges, however not in a continuous way (62). Certain LP components were also investigated in a large population and showed MBL levels are reduced in adults when compared to children (63) Previously, in a cohort of centenarians, MBL levels were reported to be reduced compared to the general population (64). However, this reduction was based on a higher prevalence of *mbl2* gene mutations, suggesting a beneficial role of intermediate levels of MBL for longevity. In our study, we found that age had a minor effect on MBL-LP activity, but significantly enhanced the activity of CP and AP. Accordingly, in an earlier study in healthy subjects aged between 20 and 69 years old, increased CH50 activity during aging was observed and associated with increased levels of individual components of the CP (65). Moreover, in line with the enhanced functional activity of CP and AP, the levels of terminal pathway components C5, C8, and C9 also raised with age. However, an important limitation of our results is that our study design is cross-sectional and not longitudinal. Accordingly, our study is unable to discriminate between true age-related changes or better survival due to evolutionary advantages. Possible explanations for the aging immune system could be epigenetic changes, metabolic changes, changes in protein production and degradation and accumulation of senescence cells. Finally, the increased levels of terminal pathway components with age could be a mechanism to compensate for the impaired clearance of pathogens and apoptotic cells due to lower cellular immunity (66).

The current findings help to become conscious of changes in complement function during physiological conditions. Furthermore, the data obtained provide insight in the complex teamwork of all the different complement proteins to achieve the end goal, i.e., the production of the membrane attack complex upon complement activation. To further study the contribution of each individual component to their respective pathway(s) we first correlated individual complement components with their complement pathway function. However, since complement components also correlated with each other, this could cause a potential confounder. Thus, we next performed multivariate regression analysis, to correct for other components and confounders. In this analysis, MBL and MASP-2 levels were independent determinants of MBL-P activity. For the CP activity, C2 and C5 levels were the strongest determinants. In accordance, C2 has previously been described as the rate-limiting factor in the CP activation (67). In addition, C1-INH was also an independent determinant of CP activity, however with a negative value, confirming that C1-INH acts as a negative regulator of CP activation (68, 69). Furthermore, AP activity was dependent on C3, factor B and C5. Factor D has previously been described as the rate-limiting step of the AP, however the current study does not confirm these results (67, 70). Our studies confirm the key role of C5 as a rate limiting step for activation of the terminal pathway of complement, both via the CP and via the AP.

Expression of complement genes and complement function is at least partially genetically determined. In the present study, the function of the MBL pathway was strongly determined by the concentration of MBL, of which the expression of functional molecules is largely genetically controlled by a number of SNP in the promoter and coding region (45, 71). Our data also show that the concentration of factor B is positively correlated to the concentrations of C4 (*r* = 0.39) and C2 (*r* = 0.42), suggesting co-regulation of the genes involved, which are located in close proximity to each other in the MHC class III region at chromosome 6 (72). Similarly, expression of C5 and C8 is strongly correlated, suggesting co-regulation of expression of genes for C5 and C8-gamma, located at chromosome 9q33.

The present study focused on complement function and individual component concentration. It was out of the scope to investigate complement activation products reflecting basic ongoing *in vivo* complement activation. In contrast to differences found in native component concentrations, we have previously shown that a number of complement activation products did not differ between female and male blood donors indicating that the degree of physiologic activation not necessarily is reflected by the different individual component concentrations (73). Furthermore, the concentration of activation products did not differ between the age groups divided in decades from 20 to 70 years (unpublished data). Others have shown that there is an individual diurnal variation of complement components and anaphylatoxins dependent of sleep (74).

We acknowledge that the present study has limitations. Although our cohort includes a variety of subjects in different age ranges, one limitation is the absence of age below and above blood donor acceptance (i.e., 20 and 69 years). Additionally, we did not have genetic and lifestyle background from the subjects of the study, which could add valuable information. However, all donors were accepted as blood donors according to the health criteria for donating blood at the hospital, implying that they represent the health part of the population. Furthermore, the complement system comprises over more than 50 proteins and we were therefore unable to determine all complement proteins. Concerning LP, the current study only measured functional activity of MBL and not from the other initiators such as ficolins and collectins. Finally, the standards used in the different assays were not calibrated to the official European complement standard. However, this would not change our conclusions, just the absolute values. On the other hand, strengths include the number of complement proteins determined, the combination of quantitative and functional analysis, the population size and the in-depth performed statistical analysis.

In conclusion, there are important sex- and age-related differences in the complement system. These changes should be taken into account when studying complement-related diseases. Furthermore, complement therapies that are now reaching the clinical phase should be tested for the different sexes and age ranges since different complement profiles might affect the efficiency of a therapy.

## Ethics statement

The study was approved by the local ethical committee of the North-Norwegian Health Authority. The Blood donors were recruited from the Bloodbank and signed a written informed consent before blood donation.

## Author contributions

Research idea and study design by MS, AR, MD, CvK; data acquisition by MS, AR, TM, FT, RW, LAT and LeT; data analysis/interpretation by MG, FP, MS, and AR. Statistical analysis by FP, MG; FP, MG, and MS wrote the manuscript. All authors were involved in editing the final manuscript. All authors read and approved the final manuscript.

### Conflict of interest statement

The authors declare that the research was conducted in the absence of any commercial or financial relationships that could be construed as a potential conflict of interest.

## Abbreviations

aHUS: Atypical haemolytic uremic syndrome
AMD: Age-related macular degeneration
AP: Alternative pathway
C1-INH: C1-inhibitor
C4bp: C4b-binding protein
C3G: C3 glomerulopathy
CP: Classical pathway
IQR: Interquartile range
LP: Lectin pathway
MAC: Membrane attack complex
MASP: MBL-associated serine protease
MBL: Mannan-binding lectin
MBL-LP: MBL-induced lectin pathway
PNH: Paroxysmal nocturnal hemoglobinuria
SLE: Systemic lupus erythematosus
SNP: Single-nucleotide polymorphism
TP: Terminal pathway.
